# Valence band structure and charge distribution in the layered lanthanide-doped CuCr_0.99_Ln_0.01_S_2_ (Ln = La, Ce) solid solutions

**DOI:** 10.1038/s41598-021-98350-9

**Published:** 2021-09-23

**Authors:** E. V. Korotaev, M. M. Syrokvashin, I. Yu Filatova, A. V. Kalinkin, A. V. Sotnikov

**Affiliations:** 1 Nikolaev Institute of Inorganic Chemistry, Siberian Branch of Russian Academy of Sciences, Novosibirsk, Russia; 2 Boreskov Institute of Catalysis, Siberian Branch of Russian Academy of Sciences, Novosibirsk, Russia

**Keywords:** Condensed-matter physics, Materials for energy and catalysis, Theory and computation

## Abstract

The comprehensive study of the electronic density distribution of CuCr_0.99_Ln_0.01_S_2_ (Ln = La, Ce) solid solutions was carried out using both X-ray photoelectron and emission spectroscopy. It was found that cationic substitution of chromium with lanthanum or cerium atoms does not significantly affect the atomic charges of the matrix elements (Cu, Cr, S) in the lanthanide-doped solid solutions. The copper atoms in the composition of CuCrS_2_-matrix and the lanthanide-doped solid solutions were found to be in the monovalent state. The chromium and lanthanide atoms were found to be in the trivalent state. This fact indicates the isovalent cationic substitution character. The sulfur atoms were found to be in the divalent state. The near-surface layers contain the additional oxidation forms of sulfur (S^0^, S^4+^_,_ S^6+^) and copper (Cu^2+^) atoms. The detailed analysis of the valence band structure using DFT calculations has shown that partial DOS distribution character of the matrix elements is preserved after the cationic substitution. The experimental valence band spectra structure of CuCrS_2_-matrix and CuCr_0.99_Ln_0.01_S_2_ is determined by the occupied copper *d*-states contribution. The contribution of the lanthanide states in the valence band structure is lower in comparison with those for the matrix elements. The major contribution of the lanthanide states was found to be mainly localized near the conduction band bottom.

## Introduction

Waste heat accounts for roughly half of total energy generation. The harvested waste heat can be cost-effective converted into electricity using the thermoelectric generators. Thus, one of the main trends of the modern material science is development of highly efficient thermoelectric materials for waste heat recovery^1,2^. The energy conversion efficiency is quantified by a thermoelectric figure of merit ZT = S^2^σT/κ, where S, σ, T, and κ are the Seebeck coefficient value, electrical conductivity, temperature, and thermal conductivity, respectively. The efficient thermoelectric material should have high values for both Seebeck coefficient and electrical conductivity and low thermal conductivity values. The combination of the corresponding parameters allows one to consider the layered dichalcogenides MCrX_2_ (M = Cu, Ag; X = S, Se) as a promising functional materials for thermoelectric applications^3–9^. These compounds are formed by alternating metal and chalcogenide layers. The unstable dichalcogenide CrX_2_-layers are stabilized by metal atoms M, intercalated between the layers. The layered structure leads to the difference between their electron and phonon mean free paths and, thereby, the thermal conductivity decrease and the electrical conductivity increase. The chromium atoms in the dichalcogenide layers could be substituted with other transition metal atoms over a wide range of concentration without spatial group change^9^. The cationic substitution of MCrX_2_-matrix with heavy 3*d-* or 4f.*-*metal atoms allows one to decrease the thermal conductivity due to the phonon scattering increase. It was reported, that cationic substitution of the CuCrS_2_ layered dichalcogenide is an effective approach for improving the material physical properties^4,7,9,11^. In particular, the substitution of chromium with iron atoms at low-level doping concentration (x ≤ 0.03) results a significant increase of the Seebeck coefficient value in comparison with those for the initial CuCrS_2_-matrix^9^. Contrariwise, an increase of iron dopant concentration suppresses the thermoelectric properties of the cation substituted solid solutions due to the metal–insulator transition (MIT). Thus, the solid solutions low-level doping concentration are of special interest. The heavy lanthanide atoms could be used to increase the phonon scattering due to the increase of the nanoscale inhomogeneity that, thereby, decrease the material thermal conductivity^1,2^. The doping of CuCrS_2_-matrix with lanthanide ions leads to the Seebeck coefficient value increase^11^. Thus, the combination of the above mentioned approaches could be considered as a promising way to optimize the thermoelectric efficiency of CuCrS_2_-based solid solutions.

Note that ZT is determined mainly by the Seebeck coefficient value. Seebeck coefficient is complicated parameter determined by both the electron density of states (DOS) distribution and the carrier concentration and its mobility^9,10^:

1 $$\mathrm{S}=-\frac{{k}^{2}}{\mathrm{e}}\frac{1}{\mathrm{n}{\upmu }_{\mathrm{n}}+\mathrm{p}{\upmu }_{\mathrm{p}}}\left\{\left[2-\frac{{\mathrm{E}}_{\mathrm{F}}}{\mathrm{kT}}\right]\mathrm{n}{\upmu }_{\mathrm{n}}-\left[2-\frac{{\mathrm{E}}_{\mathrm{F}}+{\mathrm{E}}_{\mathrm{g}}}{\mathrm{kT}}\right]\mathrm{p}{\upmu }_{\mathrm{p}}\right\}=-\frac{\mathrm{k}}{\mathrm{e}}\left\{\frac{\left[2+\mathrm{ln}\left(\frac{\mathrm{Nc}}{\mathrm{n}}\right)\right]\mathrm{n}{\upmu }_{\mathrm{n}}-\left[2+\mathrm{ln}\left(\frac{\mathrm{Nv}}{\mathrm{p}}\right)\right]\mathrm{p}{\upmu }_{\mathrm{p}}}{\mathrm{n}{\upmu }_{\mathrm{n}}+\mathrm{p}{\upmu }_{\mathrm{p}}}\right\},$$

where *k—*the Boltzmann constant, *e—*the electron charge, *n* and *p—*the concentration of electrons and holes respectively, *µ*_*n*_ and *µ*_*p*_*—*the mobility of the electrons and holes respectively*, E*_*g*_*—*the band gap width, *E*_*F*_*—*the Fermi energy, *N*_*c*_ and *N*_*v*_*—*effective DOS of the conduction and valence band respectively*.* According Eq. (1), one can conclude that the Seebeck coefficient of a *p*-type semiconductor is determined mainly by the valence band DOS as well as the Fermi level energy. The CuCrS_2_-based solid solutions are considered as *p*-type semiconductors^3–6,9,11^. Hence, the study of the electron density distribution in the valence band is of special interest.

The Seebeck coefficient of CuCrS_2_-based solid solutions is significantly changed due to the reconfiguration of the electronic structure across the MIT. This fact allows one to conclude that the partial density of states (pDOS) localization character and, thereby, the integral charge distribution are the key aspects of the interpretation and prediction of the thermoelectric properties. However, the electronic structure features in the valence band of the lanthanide-doped CuCr_0.99_Ln_0.01_S_2_ (Ln = La, Ce) solid solutions has not been discussed yet. The X-ray photoelectron (XPS) and X-ray emission spectroscopy (XES) are the most effective experimental techniques to study the electronic structure^12,13^. The combination of these techniques allows one to investigate the atom oxidation state in the near-surface layers (XPS) and in the bulk (XES) of CuCr_0.99_Ln_0.01_S_2_ (Ln = La, Ce). The partial contributions in the electronic structure features in the valence band (VB) could be studied using the density functional theory (DFT) simulation of the experimental XPS VB spectra structure.

## Methods

The powder samples CuCrS_2_ and CuCr_0.99_Ln_0.01_S_2_ (Ln = La, Ce) were synthesized from the initial metal oxides (CuO, Cr_2_O_3_, La_2_O_3_, CeO_2_) with purity of 99.99% using procedure described and reported previously in^11^. The current research was carried out using the samples from our previous work^11^. The synthesized samples were reported to be single-phase and isostructural to the initial CuCrS_2_-matrix^11,13^. The obtained crystal structure data agree well with the crystallographic data reported in^14^.

The XPS lines were measured using a SPECS spectrometer with a PHOIBOS-150 hemispherical analyzer. The sulfur (S2*p*), copper (Cu2*p*), lanthanides (Ln3*d*) lines and valance band spectra were recorded using Al*K*α radiation (E = 1486.6 eV). The chromium (Cr2*p*) lines were recorded using Mgα radiation (E = 1253.6 eV). The residual pressure in the spectrometer analysis chamber was < 1·10^−9^ Torr. The samples were fixed on a sample holder using a conductive double sided adhesive carbon tape and held at room temperature during the measurements. The XPS spectra processing (decomposition into individual components, measurement of XPS signal area and binding energies) was carried out after the background subtraction using CasaXPS 2.3^15^. The experimental spectra were corrected with Shirley background approximation and fitted with the Gaussian–Lorentzian function. The binding energy scale was calibrated with the internal standard method using the C1*s* line (284.8 eV) for the carbon contained on the sample surface. The measurement accuracy of XPS binding energy was 0.2 eV.

The X-ray emission spectra (XES) of sulfur (S*Kα*), chromium (Cr*Kα*) and copper (Cu*Kα*) were measured using Johann-type X-ray spectrometer with a cylindrically bent quartz $(10\overline{1}1)$ crystal-analyzer (X-ray tube voltage V = 24 kV, current I = 15 mA). The base pressure in the spectrometer analysis chamber was ~ 2.5·10^−5^ Torr. The samples on a double sided adhesive carbon tape were fixed on a secondary aluminum anode and held at a liquid nitrogen temperature during the measurements. The spectra were measured using a gas-flow argon-methane proportional counter. The measurement accuracy of the S*K*α_1,2_- and Cr*K*α_1,2_-lines energy position was 0.05 eV, for the Cu*K*α_1,2_-lines was 0.1 eV. The reference compounds Cu_2_S (99.99%), Cr_2_O_3_ (99.9%) and KSCN (≥ 99.0%) used are the commercial chemicals (Millipore Sigma, USA). The spectrometer resolution in the spectral region being studied was ~ 5·10^−4^. The XES spectra processing was carried out using XPSPeak fitting program^16^.

The partial density of states (pDOS) distribution in the valence band was calculated in BAND software package^17^ using the generalized gradient approximation (GGA), the standard Slater-type orbital basis set using three basis function per atomic orbital with one polarization function (TZP) and the Perdew*-*Burke*-*Ernzerhof exchange–correlation potential (PBESol-D). The crystallographic data for initial CuCrS_2_-matrix were taken from the Inorganic Crystal Structure Data base (ICSD)^14^. In the case of the lanthanide-doped CuCr_1-x_Ln_x_S_2_ (Ln = La, Ce) solid solutions one of the three chromium atoms in the unit cell CuCrS_2_ was replaced by lanthanum or cerium atom (x≈0.33) and the geometry was optimized.

The experimental XPS valence band spectra were compared with calculated data taking into account both the photoionization cross sections and atomic concentrations^18^. The calculated pDOS were broadened in order to take into account the AlKα-line width and the instrumental resolution using a Lorentz-type distortion function with half-width of 1 eV^19^.

The electrical resistivity temperature dependencies were measured in an atmosphere of helium at a pressure reduced to 5 Torr on the cylindrical compressed (~ 70 MPa) samples using two-probe resistance measurement technique. The samples were compressed in vacuum at 650 °C. The estimated sample density was ~ 4.1 g/cm^3^. The Thermodat-13K5 temperature controller was used for the temperature control and stabilization. The electrical resistivity was measured using digital multimeter Keysight 34461A.

## Results and discussion

**Figure 1:**
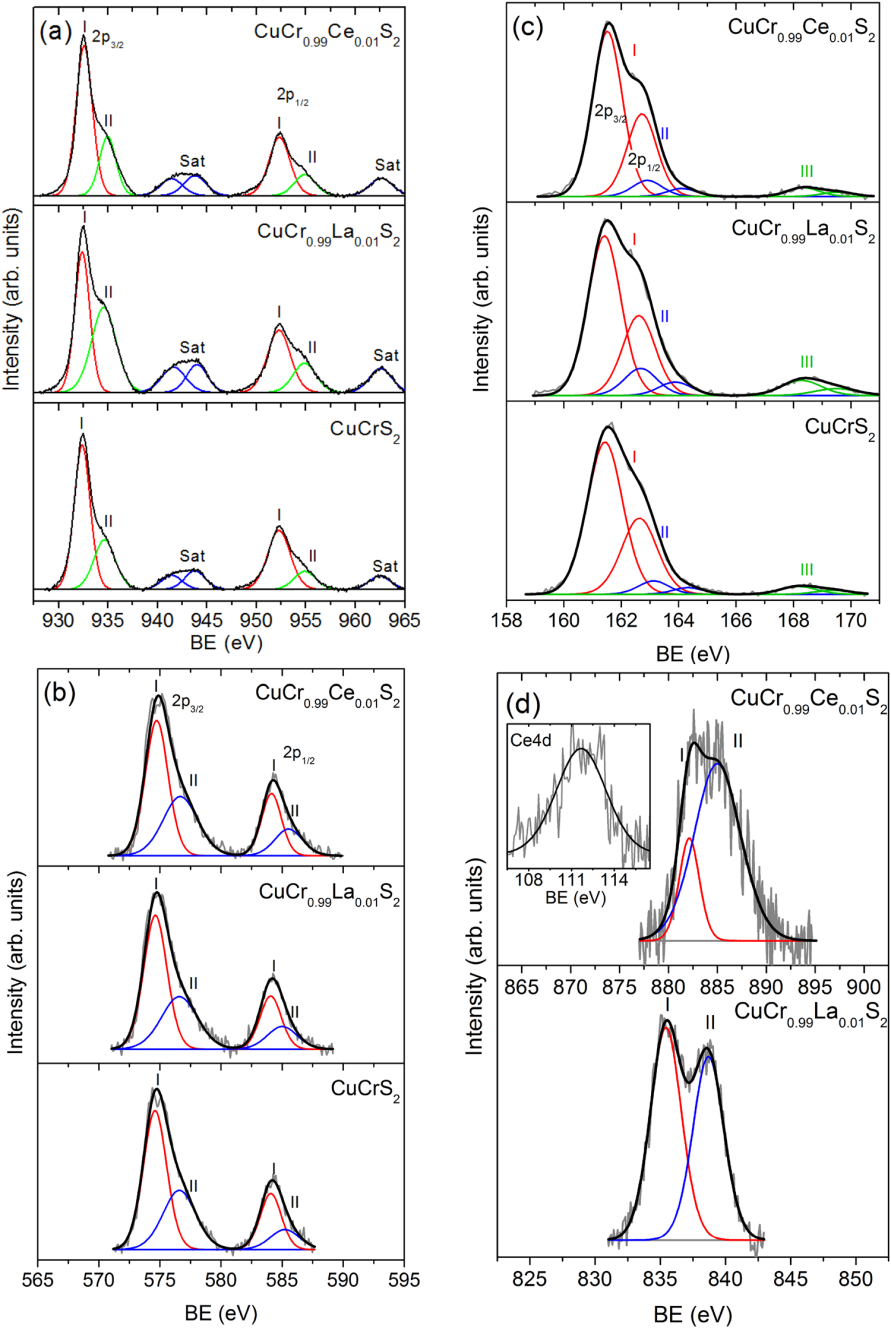
XPS lines of CuCrS_2_-matrix and lanthanide-doped solid solutions CuCr_0.99_Ln_0.01_S_2_ (Ln = La, Ce): (**a**) Cu(2*p*_*3/2*_)-, (**b**) Cr(2*p*_*3/2*_)-, (**c**) S(2*p*)- and Ln(3*d*_*5/2*_)-lines (**d**). Inset: Ce4*d*-region.

The XPS data allows one to determine the binding energy (BE) of individual core levels. The core level energy depends on the charge located on the constituent atoms. The charge redistribution affects the core level energy. Thus, XPS S(2*p*)-, Cr(2*p*)-, Cu(2*p*)- and La, Ce(3*d*)-core level were recorded to determine the atom oxidation state (Fig. 1).

Figure 1a shows the XPS Cu(*2p*_*3/2,1/2*_)-lines of CuCr_0.99_Ln_0.01_S_2_ (Ln = La, Ce) powder samples. The Cu(2*p*)-region exhibits two intense peaks corresponding to 2*p*_3/2_ and 2*p*_1/2_ levels (~ 932 and ~ 952 eV, respectively) and two groups of the satellite lines *Sat* (~ 943–946 and ~ 960–965 eV, respectively).

The satellite lines are characteristic for Cu^2+^ compounds and arising due to shake-up states corresponding to the *p*^5^*d*^9^ electronic configuration^20^. The Cu(2*p*_*3/2*_)- and Cu(2*p*_*1/2*_)-lines could be represented as a superposition of lines arising from the nonequivalent copper atoms (marked as *I* and *II* in Fig. 1) related to the different oxidation states^21–23^. The copper oxidation state analysis was carried out using the binding energy (BE) value of Cu(2*p*_*3/2*_)-lines (Table 1). The BE value of low energy *I* component (~ 932.5 eV) is typical for the compounds containing Cu^+^ (BE(Cu_2_S) ≈932.3 eV; BE(Cu_2_O) ≈932.4 eV; BE(CuCl) ≈932.3 eV)^19,24,25^. The BE of the high energy *II* component (~ 934.7 eV) is typical for Cu^2+^ compounds (BE(CuO) ≈933.7 eV; BE(CuCl_2_) ≈933.7 eV; BE(CuSO_4_) ≈935.4 eV)^19,24,25^. The high energy components *II* are assumed to include the defected near-surface layers^22,26,27^. These layers contain oxygen-contained compounds of Cu^2+^. This fact is additionally approved with data reported on the vanadium-doped CuCr_1-x_V_x_S_2_ solid solutions^13,22,23^.

**Table 1:** Binding energies values (eV) of the XPS Cu(2*p*_3/2_)-, Cr(2*p*_3/2_)-, S(2*p*_3/2_)-, La(3*d*_5/2_)- and Ce(3*d*_5/2_)-lines.

Compound	Cu(2*p*_3/2_)	Cr(2*p*_3/2_)	S(2*p*_3/2_)	Ln(3*d*_5/2_)
CuCrS_2_	932.4	574.6	161.4	–
	934.6	576.6	163.1	–
	–	–	168.2	–
CuCr_0.99_La_0.01_S_2_	932.4	574.6	161.5	835.4
	934.6	576.6	162.9	838.6
	–	–	168.3	–
CuCr_0.99_Ce_0.01_S_2_	932.6	574.7	161.5	882.1
	934.8	576.7	162.9	886.3
	–	–	168.3	–

The core-level X-ray emission spectroscopy (XES) as well as XPS could be sufficiently used to determine the effective atomic charges in molecules and solids. The main difference between these two experimental techniques is that in contrast to XPS, XES allows one to determine the atom oxidation state in the in the bulk. Thus, the combination of both XPS and XES allows one to obtain the total data on the atomic charges in CuCr_0.99_Ln_0.01_S_2_ (Ln = La, Ce).

**Figure 2:**
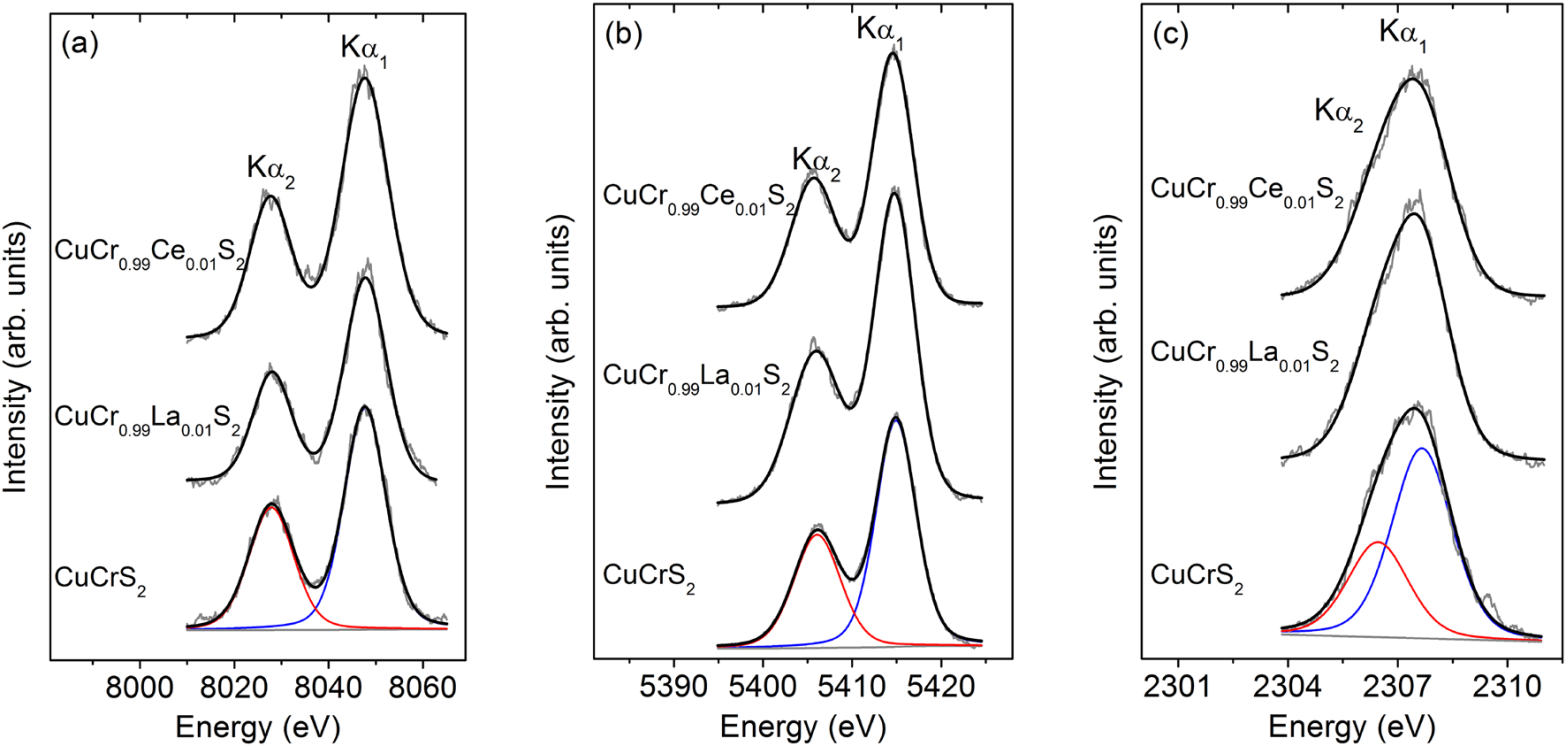
XES Kα_1,2_-spectra of CuCrS_2_-matrix and CuCr_0.99_Ln_0.01_S_2_ (Ln = La, Ce) solid solutions: (**a**) Cu*Kα*_*1,2*_-, (**b**) Cr*Kα*_*1,2*_- and (**c**) S*Kα*_*1,2*_-spectra.

The XES Cu*K*α_*1,2*_-spectra for CuCrS_2_-matrix and lanthanide-doped CuCr_0.99_Ln_0.01_S_2_ (Ln = La, Ce) solid solutions are depicted in Fig. 2a. The Cu*K*α-region exhibits two intense peaks corresponding to the 2*p*_3/2,1/2_ → 1* s* transitions. The line-shape of the Cu*K*α_*1,2*_-spectra does not significantly change after the cationic substitution. Note that additional oxidation forms of copper were not observed in the Cu*K*α_*1,2*_*-*region. This fact indicates that Cu^2+^ forms arising in the Cu2*p*-region are contained only in the defected near-surface layers. The energy position of the Cu*K*α_*1*_-line maximum for CuCr_0.99_Ln_0.01_S_2_ lies within the range of 8047.7–8047.8 eV (Table 2). The measured values are typical for Cu^+^ atoms and agree well with one for Cu_2_S reference compound containing monovalent copper atom. Thus, one can conclude that the copper atomic charge is preserved after cationic substitution of CuCrS_2_-matrix with Ce and La atoms.

**Table 2:** Energy position of X-ray copper, chromium and sulfur *Kα*_*1,2*_*-*emission lines maximum (eV).

Compound	Cu(*Kα*_*1*_)	Cr(*Kα*_*1*_)	S(*Kα*_*1*_)
CuCrS_2_	8047.7	5414.95	2307.36
CuCr_0.99_La_0.01_S_2_	8047.8	5414.98	2307.34
CuCr_0.99_Ce_0.01_S_2_	8047.8	5414.90	2307.35
Cu_2_S	8047.9	–	–
Cr_2_O_3_	–	5414.94	–
KSCN	–	–	2307.60

The XPS Cr(2*p*_*3/2,1/2*_)-lines are depicted in Fig. 1b. The Cr(2*p*)-region exhibits two intense 2*p*_*3/2*_ and 2*p*_*1/2*_ spin doublet components (~ 575 and ~ 584 eV, respectively). The main 2*p*_*3/2*_*-*line could be presented as a superposition of two components (*I* and *II*) related to the different oxidation states. The BE of the low energy *I* component (~ 574.7 eV) for Cr(2*p*_*3/2*_)-line is typical for the compounds containing Cr^3+^ (BE(Cr_2_S_3_) ≈575.2 eV; BE(CuCrSe_2_) ≈574.7 eV; BE(CuCr_2_Se_4_) ≈574.5 eV)^19,24,25^.

The high energy components *II* (~ 576.6 eV) could be related to the oxygen-containing compounds of Cr^3+^ on the powder surface (BE(Cr_2_O_3_) ≈576.5 eV; BE(CuCrO_2_) ≈576.0 eV)^19,24,25,28^. It should be noted that the measured BE values of Cr(2*p*_*3/2*_) are in good agreement with previously reported XPS data for CuCrS_2_-matrix^5,21–23,29^. Figure 2b plots the XES Cr*K*α_1,2_-region of CuCrS_2_-matrix and lanthanide-substituted solid solutions CuCr_0.99_Ln_0.01_S_2_ (Ln = La, Ce). The energy position of chromium *Kα*_*1*_-maxima lies within the range of 5414.90–5414.98 eV (Table 2). Taking into account the measurement accuracy, the Cr*Kα*_*1*_-maxima energy position values correlate with one for Cr_2_O_3_ reference compound and typical for Cr^3+^^13^. The absence of the significant chemical shifts of Cr(*2p*)- and Cr*Kα*-lines indicates that cationic substitution does not significantly affect the chromium atomic charge. The Cr*Kα*-line shape fact indicates the absence of the additional chromium oxidation states. Note, that *2p* to *1 s* core level electron transition, corresponding to XES *Kα*-lines, occurs in the Coulomb field created by surrounding atoms of the system. Thus, the position of the XES line depends only on the electron density localized on the investigated atom. The potential from the chemical surrounding does not affect the XES lines energy position. Hence, the additional components arising in Cr(*2p*)-region from the Cr^3+^ oxidation forms could not be resolved in Cr*Kα*-region ^13^.

Figure 1c plots the XPS S(2*p*_*3/2,1/2*_)-lines of CuCrS_2_-matrix and lanthanide-substituted solid solutions. Table 1 lists the experimental BE values of S(2*p*_*3/2*_)-lines. The S(2*p*)-line is a superposition of two components (S(2*p*_*1/2*_) and S(2*p*_*3/2*_)) arising from the spin–orbit coupling of S2*p* core-level. The experimental S(2*p*)-region presents a superposition of lines arising from the several nonequivalent groups containing different types of sulfur atoms (denoted as *I*, *II* and *III* in Fig. 1c).

The first group (*I*) includes the sulfur atoms in the composition of the CuCrS_2_-matrix and lanthanide-doped solid solutions (Table 1). The energy position of S(*2p*_3/2_)-line (BE ~ 161.5 eV) corresponds to S^2−^ oxidation state and is typical for the transition metal sulfides (BE(CuFeS_2_) ≈161.5 eV; BE(Cu_2_S) ≈161.8 eV)^19,24,25^. The second group of S(2*p*)-lines (*II*) with BE ~ 163 eV is assumed to include the elemental sulfur (BE ≈ 163.5 eV) on the powder surface. The presence of the “surface” elemental sulfur is typical for the natural and synthesized sulfide materials^26^. The last group of S(2*p*)-lines (*III*) with BE ~ 168.2 eV is assumed to include the oxygen-containing compounds of sulfur on the powder surface (BE(CuSO_4_) ≈169.6 eV; BE(C_12_H_8_SO_2_) ≈168.2 eV, BE ((NH_4_)_2_SO_4_) ≈168.3 eV; BE(SO(CH_3_O)_2_) ≈168.4 eV)^19,24,25^.

The XES S*Kα*_*1,*2_-spectra are unresolved spin-doublets (Fig. 2c). The measured S*K*α_1_-line energy position values correspond to divalent sulfur and correlates with one for KSCN reference compound containing S^2−^ (Table 2). The S*Kα*_*1,2*_-line shape and the energy position allow one to conclude that additional groups of sulfur atoms are localized in the near-surface layers. Thus, the absence of the significant chemical shifts of the S(2*p*)- and S*K*α-lines allow one to conclude that cationic substitution of CuCrS_2_-matrix with lanthanide atoms does not significantly affect the sulfur atoms oxidation state.

The XPS lines of lanthanides (La and Ce) are presented in Fig. 1d. The BE values of Ln(3*d*_*5/2*_)-lines are listed in Table 1. The Ln(3*d*)-region exhibits the main peak (*I*) accompanied with a satellite line (*II*) arising due to the multi-electron processes (multiplet structure)^30^. The La(3*d*_*5/2*_)-line is unresolved peak with structure typical for La^3+^ (two components with the similar intensity)^24^. The obtained La(3*d*_*5/2*_) BE value equal to ~ 835.4 eV corresponds to La^3+^ (BE (La_2_O_3_) ≈835 eV)^24,25^.

The multiplet splitting (MS) between the main and the satellite lines in the Ln(3d)-spectra allows one to identify the Ln oxidation state. The measured MS value of La(3d5/2)-line is equal to 3.2 eV. The typical MS value for La^3+^ compounds lies within the range of ~ 3.5–4.6 eV^25^. Thus, one can conclude that lanthanum atoms in the composition of CuCr_0.99_La_0.01_S_2_ are trivalent. In contrast to the lanthanum, the cerium could exhibit a few stable oxidation forms (Ce^3+^ or Ce^4+^). It should be noted that as it was reported previously for vanadium-doped solid solutions CuCr_1-x_V_x_S_2_ (x < 0.15), the vanadium atoms were found to be in V^4+^ oxidation state^13^. Hence, the cerium oxidation state is of special interest. The Ce(3*d*_*5/2*_)-line has a structure similar as those for La(3*d*_*5/2*_)-line. The BE values corresponding to the different oxidation forms of the cerium atoms lies almost in the same energy region. For instance, the BE value for Ce^4+^ (BE(Ce^4+^) ≈882–882.7 eV) is slightly higher than for Ce^3+^ (BE(Ce^3+^) ≈880–881.5 eV)^24,25,31,32^. The measured BE value of Ce(3*d*_*5/2*_)-line for cerium-doped solid solution CuCr_0.99_Ce_0.01_S_2_ is equal to ~ 882.1 eV. At the same time, the MS values for Ce^4+^ and Ce^3+^ are reported to have more significant difference (MS(Ce^4+^) ~ 6.3 eV; MS(Ce^3+^) ~ 4.5 eV)^24,25,31,32^. Allowing for this, the Ce(3*d*_*5/2*_)-line multiplet structure analysis could provide more reliable information on the cerium oxidation state. The measured MS value for CuCr_0.99_Ce_0.01_S_2_ is equal to ~ 4.2 eV. Thus, taking into account the preservation of the Cu, Cr and S atomic charges discussed above, one can conclude that Cr^3+^ is substituted by Ce^3+^. This fact was additionally approved by the BE value of ~ 112 eV for Ce(4*d*)-spectra, typical for Ce^3+^ oxidation state^33,34^.

Thus, both XPS and XES spectra analysis have shown that doping of CuCrS_2_-matrix with lanthanides does not significantly change the atomic charges of the matrix elements. The chromium and lanthanide atoms in the composition of CuCr_0.99_Ln_0.01_S_2_ (Ln = La, Ce) were found to be in trivalent oxidation state. The cationic substitution does not affect the copper and sulfur atoms oxidation state. The copper atoms oxidation state remains monovalent (Cu^+^), the sulfur*—*divalent (S^2−^). The results obtained are well agreed to the previously reported copper, chromium and sulfur XANES investigation data^11^. Note that the high sensitivity of the XPS spectroscopy allowed one first time study the lanthanide atoms oxidation state in CuCr_0.99_Ln_0.01_S_2_ (Ln = La, Ce).

The near-surface layers of the sample studied were found to contain additional oxidation forms of copper (Cu^2+^) and sulfur (S^0^, S^4+^, S^6+^). The presence of the additional copper oxidation form (Cu^2+^) indicates that the surface layers contain the additional scattering centers. These centers are assumed to increase the electrical resistance component related to the carrier scattering on the material “magnetic structure". The grain boundary magnetic scattering is one of the significant aspects in the interpretation of the colossal magnetic resistance (CMR) origin^35^. Thus, the electronic density distribution study of CuCrS_2_ and CuCr_0.99_Ln_0.01_S_2_ allows one to expect the promising CMR values due to the presence of the Cu^2+^ oxidation states on the powder surface. It should be noted that the presence of the additional oxidation forms of sulfur and copper atoms in the composition of the oxygen-containing compounds on the powder surface should significantly affect the thermoelectric properties. The oxygen-containing compounds typically have band gaps on the order of 2 to 4 eV. For instance, CuO and CuSO_4_ have band gaps of ~ 1.5 and ~ 4 eV, respectively^36,37^. Thus, their presence on the powder surface could increase the Seebeck coefficient value of CuCrS_2_-based solid solutions. This conclusion correlates with the fact that the Seebeck coefficient value for CuCrS_2_-matrix crystal samples were reported to be two times lower than one for ceramic and compacted powder samples^5,9^.

The physical properties of chemical compounds are determined by the electronic structure features. The data on the electron density and partial orbital contributions in the band structure could be studied using the combination of both experimental and theoretical approaches. Thus, the current study involves the DFT calculation of the partial density of states (pDOS) and the experimental XPS valence band (VB) investigation.

**Figure 3:**
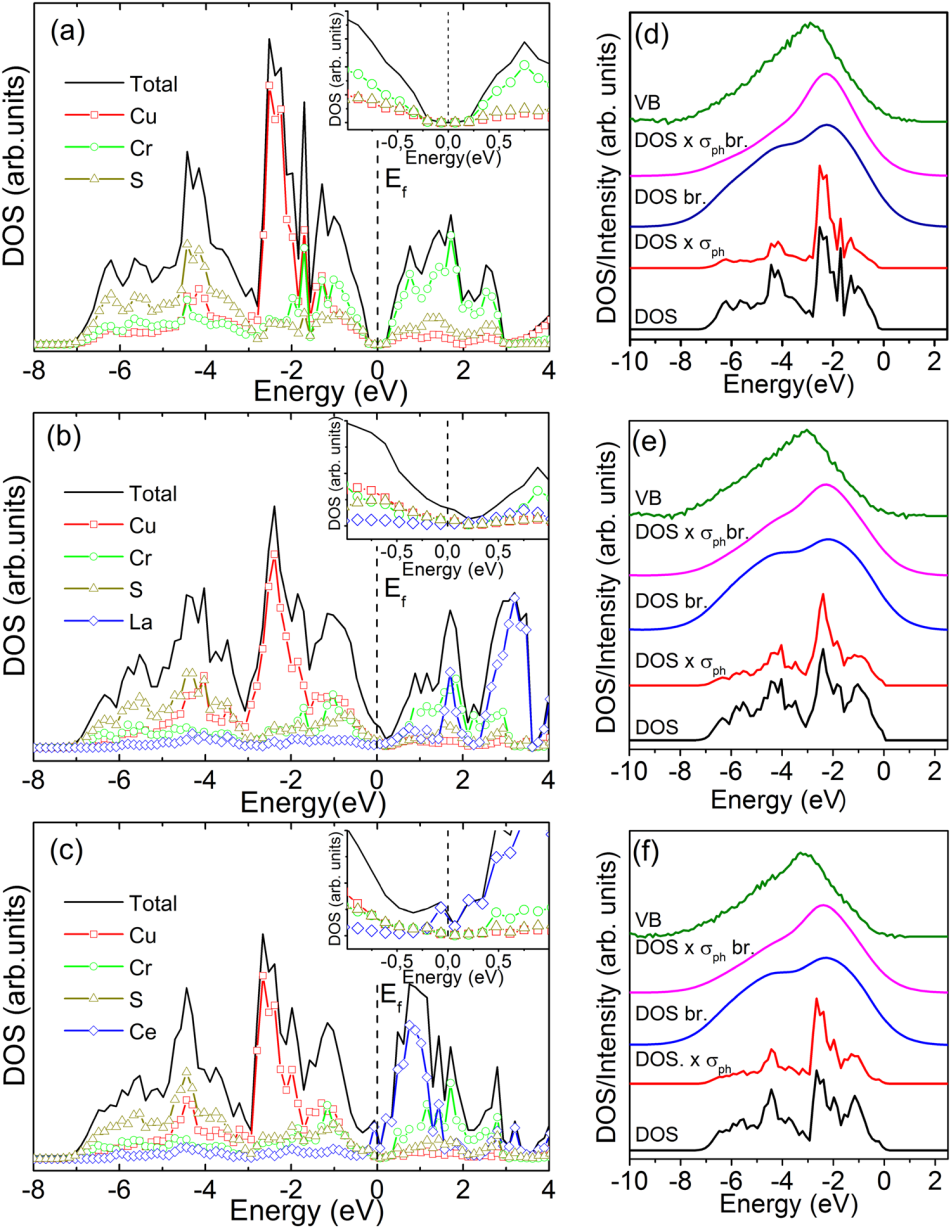
Theoretical total and partial DOS (**a**–**c**) for CuCrS_2_ and CuCr_0.99_Ln_0.01_S_2_ (Ln = La, Ce). Comparison of the experimental valence band (VB) spectra (green line) with theoretical DOS simulations (**d**–**f**) showing raw DOS simulations (black line); simulated DOS mathematically broadened for better consistency with the experimental VB (blue line); DOS scaled to the photoionization cross-sections of respective levels (red lines); DOS scaled to the photoionization cross-sections of respective levels; scaled and broadened DOS (magenta line).

The pDOS of CuCrS_2_-matrix and lanthanide-doped CuCr_1-x_Ln_x_S_2_ (Ln = La, Ce) solid solutions are presented in Fig. 3a–c. The electronic structure of CuCrS_2_-matrix is shown in Fig. 3a. The main chromium and copper *d*-states contributions are located near the valence band top at − 1 and − 2.5 eV below the Fermi level, respectively. The sulfur *p*-states contribution is mostly localized at − 4 eV. The conduction band bottom structure is dominated by the chromium *d*-states. The sulfur *p*-states and mixed copper *p*-, *d*-, *s*-states have a smaller contribution to the conduction band bottom structure. According to the data obtained, the undoped copper-chromium disulfide is a semiconductor with a band gap of ~ 0.29 eV (inset in Fig. 3a). The calculated pDOS distribution character is in good agreement with both experimental and calculated data reported in^4,38^.

The cationic substitution of CuCrS_2_-matrix does not significantly affect the partial DOS distributions of the Cu, Cr and S matrix elements (Fig. 3b,c). Since the lanthanum has empty 4f.-shell the lanthanum states contribution in the valence band structure is dominated by d-states. The main contribution of the lanthanum occupied *d*-states is localized at ~  − 4 eV in the valence band. The lanthanum unoccupied *f*-states main contribution is localized in the conduction band at ~ 3 eV.

Since the presence of electron in 4f.-orbital, the main occupied cerium *f*-states contributions in CuCr_1-x_Ce_x_S_2_ are localized in the valence band top at ~  − 0.2 eV. The unoccupied cerium *f*-states are shifted to the conduction band bottom and localized at ~ 1 eV.

The lanthanide-doped CuCr_1-x_Ln_x_S_2_ solid solutions conduction band bottom structure is dominated by the lanthanide *f*-states. The presence of the lanthanide states causes the metal–insulator transition (MIT) and the band gap vanishing (insets in Fig. 3b,c).

The experimental XPS valence band (VB) spectra are plotted in Fig. 3d–f (green lines). The simulated DOS (black lines in Fig. 3d–f) were mathematically broadened for better consistency with the experimental VB (blue lines). However, the line shape of broadened DOS (marked with “br.” in Fig. 3d–f) overestimates the intensity of the low energy shoulder feature in comparison with those for VB. Note that the photoionization cross section value (σ_ph_) for the copper 3*d*-sates is greater in comparison with those for sulfur 3*p*- and chromium 3*d*-states. Hence, the simulated DOS were scaled taking into account the σ_ph_ of respective levels (denoted as DOS × σ_ph_ in Fig. 3d–f, red lines). Thus, the better consistency of simulated DOS with experimental VB was observed for the scaled and broadened DOS (magenta line). The experimental VB structure of CuCrS_2_-matrix is mainly determined by the contribution of the copper states localized at ~  − 2.5 eV (Fig. 3d). The simulated and experimental VB for CuCr_0.99_Ln_0.01_S_2_ (Fig. 3e,f) have a structure character similar to those for CuCrS_2_-matrix (Fig. 3d). The data on the partial density of states and the band structure features are of special interest for thermoelectric compounds. As it was mentioned above, in the terms of the band theory the Seebeck coefficient of the semiconductors could be described as a function of DOS and carrier concentration (see Eq. (1) )^10^.

The isovalent substitution character discussed above allows one to conclude that the cationic substitution of CuCrS_2_-matrix with La or Ce does not emerge the additional charge carriers. Thus, according to the Eq. (1) the Seebeck coefficient of the lanthanide-doped CuCr_0.99_Ln_0.01_S_2_ (Ln = La, Ce) solid solutions is dominated by the DOS distribution character. This fact is in good agreement with an increase of the Seebeck coefficient value for CuCr_1-x_Ln_x_S_2_ in comparison with those for CuCrS_2_-matrix^11^. The replacing of the occupied chromium 3*d*-states by the unoccupied lanthanum 4f.-states causes the electronic density decrease in the valence band region. Thus, the Seebeck coefficient value in CuCr_0.99_Ln_0.01_S_2_ increases in comparison with those for initial matrix. The lower Seebeck coefficient values for the cerium-doped solid solution is due to the presence of the electron in the Ce 4f.-orbital and the localization of the occupied cerium *f*-states in the valence band top.

**Figure 4:**
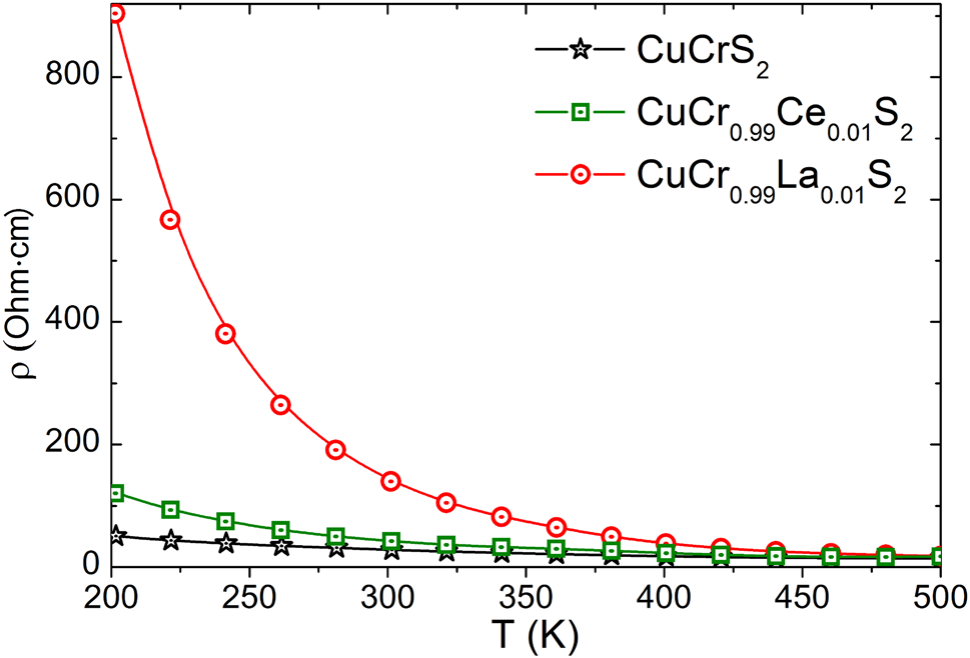
Temperature dependence of electrical resistivity for CuCrS_2_ and CuCr_0.99_Ln_0.01_S_2_ (Ln = La, Ce).

The described electronic structure features affect the electrical resistivity of the compounds studied (Fig. 4). The lanthanum-doped CuCr_0.99_La_0.01_S_2_ solid solution exhibits the highest electrical resistivity values in comparison with those for CuCr_0.99_Ce_0.01_S_2_ and CuCrS_2_-matrix. Thus, the increase of the Seebeck coefficient value is accompanied with the electrical resistivity increase. Note that the DFT calculation was carried out for the model compounds CuCr_1-x_Ln_x_S_2_ at high lanthanide concentrations (x≈0.33). The reported data on the electrophysical properties of CuCrS_2_-based solid solutions indicates that the high doping concentration (x ≥ 0.2) causes the MIT^9,39^. The MIT results the band-gap vanishing due to the presence of the additional states near the Fermi-level. Thus, the band-gap vanishing is accompanied by the electrical resistivity decreasing. In case of the CuCr_0.99_Ln_0.01_S_2_ the electrical resistivity decrease was not observed due to the low-doping concentration of lanthanide atoms. However, the DOS calculation for concentration of 0.33 allow one to predict the MIT in case of high doping level.

## Conclusion

The electronic density and the atom oxidation state comprehensive study was carried out using experimental (XPS and XES) techniques and DFT calculations. It was shown that cationic substitution does not significantly affect the atomic charges and the partial DOS distribution character on the matrix elements (Cu, Cr, S). The experimental valence band spectra structure of CuCrS_2_-matrix and CuCr_0.99_Ln_0.01_S_2_ (Ln = La, Ce) is mainly determined by the occupied copper *d*-states contribution. The contribution of the lanthanide-states in the valence band structure is lower in comparison with those for the matrix elements. The major contributions of the lanthanum and cerium electronic states were found to be localized near the conduction band bottom. It was shown that the partial DOS distribution character determines the Seebeck coefficient value increase of CuCr_1-x_Ln_x_S_2_ in comparison with those for CuCrS_2_-matrix. The lanthanum-doped CuCr_0.99_La_0.01_S_2_ solid solution exhibits the highest electrical resistivity values. The lanthanide and chromium atoms were found to be in the trivalent state, while the copper atoms in the monovalent state. The sulfur atoms in the composition of CuCrS_2_-matrix and the lanthanide-doped solid solutions were found to be in the divalent state. The near-surface layers contain the additional oxidation forms of the sulfur (S^0^, S^4+^_,_ S^6+^) and the copper (Cu^2+^) atoms.

## References

[1] Aswal DK, Basu R, Singh A (2016). Key issues in development of thermoelectric power generators: High figure-of-merit materials and their highly conducting interfaces with metallic interconnects. Energy Convers. Manag..

[2] Zhang J, Song L, Iversen BB (2020). Probing efficient N-type lanthanide dopants for Mg_3_Sb_2_ thermoelectrics. Adv. Sci..

[3] Hansen AL, Dankwort T, Groβ H, Etter M, König J, Duppel V, Kienle L, Bensch W (2017). Structural properties of the thermoelectric material CuCrS_2_ and of deintercalated Cu_x_CrS_2_ on different length scales: X-ray diffraction, pair distribution function and transmission electron microscopy studies. J. Mater. Chem. C.

[4] Srivastana D, Tewari GC, Kappinen M, Nieminen RM (2013). First-principes study of layered antiferromagnetic CuCrX_2_ (X=S, Se and Te). J. Phys. Condens. Matter..

[5] Tewari GC, Tripathi TS, Kumar P, Rastogi AK, Pasha SK, Gupta G (2011). Increase in the thermoelectric efficiency of the disordered phase of layered antiferromagnetic CuCrS_2_. J. Electron. Mater..

[6] Korotaev EV, Syrokvashin MM, Filatova IY, Sotnikov AV (2021). Effect of the order-disorder transition on the electronic structure and physical properties of layered CuCrS_2_. Materials.

[7] Bhattacharya S, Bohra A, Basu R, Bhatt R, Ahmad S, Meshram KN, Debnath AK, Singh A, Sarkar SK, Navneethan M, Hayakawa Y, Aswal DK, Gupta SK (2014). High thermoelectric performance of (AgCrSe2)0.5(CuCrSe2)0.5 nano-composites having all-scale natural hierarchial architectures. J. Mater. Chem. A.

[8] Wu D, Huang S, Feng D (2015). Revisiting AgCrSe_2_ as promising thermoelectric material. Phys. Chem. Chem. Phys..

[9] Korotaev EV, Syrokvashin MM, Filatova IYu, Pelmenev KG, Zvereva VV, Peregudova NN (2018). Seebeck coefficient of cation-substituted disulfides CuCr_1-x_Fe_x_S_2_ and Cu_1-x_Fe_x_CrS_2_. J. Electron. Mater..

[10] Shalimov KV (2021). Semiconductors Physics.

[11] Korotaev EV, Syrokvashin MM, Filatova Iu, Trubina SV, Nikolenko AD, Ivlyushkin DV, Zavertkin PS, Sotnikov AV, Kriventsov VV (2020). XANES investigation of novel lanthanide-doped CuCr099Ln001S2 (Ln = La, Ce) solid solutions. Appl. Phys. A.

[12] Syrokvashin MM, Korotaev EV, Kryuchkova NA, Zvereva VV, Filatova IYu, Kalinkin AV (2019). Surface and bulk charge distribution in manganese sulfide doped with lanthanide ions. Appl. Surf. Sci..

[13] Korotaev EV, Syrokvashin MM, Filatova IYu, Zvereva VV (2020). Vanadium doped layered copper-chromium sulfides: the correlation between the magnetic properties and XES data. Vacuum.

[14] Inorganic Crystal Structure Database. Version 2.1.0 / FIZ Karlsruhe, Germany.

[15] CasaXPS: Processing Software for XPS, AES, SIMS and More, http://www.casaxps.com/

[16] Kwok RWM (1999). XPS Peak Fitting Program for WIN95/98 XPSPEAK Version 4.1.

[17] BAND 2016, SCM, Theoretical Chemistry, Vrije Universiteit, Amsterdam, The Netherlands. http://www.scm.com

[18] Feldman LC, Mayer JW (1986). Fundamentals of Surface and Thin Film Analysis.

[19] Nefedov VA (1984). X-Ray Photoelectron Spectroscopy of Chemical Compounds.

[20] Larsson S (1976). Satellites in ESCA inner-shell spectra of 3d0 transition metal complexes. J. Electron. Spectros. Relat. Phenomena.

[21] Mazalov LN, Sokolov VV, Kryuchkova NA, Vovk EI, Filatova IYu, Abramova GM (2009). X-ray photoelectron spectroscopic studies of the charged state of 3d metal ions in CuCr1-xVxS2 (x=0–0.4). J. Struct. Chem..

[22] Korotaev EV, Peregudova NN, Mazalov LN, Sokolov VV, Kalinkin AV, Kryuchkova NA, Dikov YuP, Buleev MI, Filatova IYu, Pichugin AYu (2013). Photoelectron spectra of powder and single crystalline chromium-copper disulfides. J. Struct. Chem..

[23] Mazalov LN, Dikov YuP, Kryuchkova NA, Sokolov VV, Filatova IYu, Korotaev EA, Fedorenko AD (2010). XPS spectra of vanadium-doped disulfides CuCrS_2_. J. Struct. Chem..

[24] NIST Standard Reference Database 20, Version 4.1, 10.18434/T4T88K

[25] XPS Simplified: XPS Data Interpretation (2020). https://xpssimplified.com/whatisxps.php (accessed 18 September 2020)

[26] Mikhlin YL, Tomashevich YV, Pashkov GL, Okotrub AV, Asanov IP, Mazalov LN (1998). Electronic structure of the non-equilibrium iron-deficient layer of hexagonal pyrrhotite. Appl. Surf. Sci..

[27] Vasilyeva IG (2017). Chemical aspect of the structural disorder in CuCrS_2_ and CuCr_1-x_V_x_S_2_ solid solutions. J. Struct. Chem..

[28] Zhou S, Fang X, Deng Z, Li D, Dong W, Tao R, Meng G, Wang T, Zhu X (2008). Hydrothermal synthesis and characterization of CuCrO_2_ laminar nanocrystals. J. Cryst. Growth.

[29] Hollander JCTh, Sawatzky G, Haas C (1974). Monovalent copper in the chalcogenide spinel CuCr_2_Se_4_. Solid State Commun..

[30] Teterin YuA, Teterin AYu, Lebedev AM, Utkin IO, Nikitin AS (1998). Dynamic effect on the structure of X-ray photoelectron spectra of lanthanide fluorides and oxides. J. Struct. Chem..

[31] Heng CL, Li JT, Su WY, Yin PG, Finstad TG (2015). The photoluminescence and structural properties of (Ce, Yb) co-doped silicon oxides after high temperature annealing. J. Appl. Phys..

[32] Bêche E, Charvin P, Perarnau D, Abanades S, Flamant G (2008). Ce 3d XPS investigation of cerium oxides and mixed cerium oxide (Ce_x_Ti_y_O_z_). Surf. Interface Anal..

[33] Taheri M, Konuma M, Razavi FS (2017). X-ray photoemission spectroscopy investigation of Ce_1−x_Eu_x_CrO_3_ nano-powders. Surf. Interface Anal..

[34] Dudric, R., Souca, G., Kuepper, K. & Tetean, R, XPS on Gd_1−x_Ce_x_Co_2_ Intermetallic Compounds, Phys. Status Solidi. **256** 1800320 (2019). pssb.201800320.

[35] Shirinzadeh H (2014). The phenomenon of colossal magnetoresistance and some experimental results. Int. J. Fundam. Phys. Sci..

[36] Bakr NA, Dhahir TAA, Mohammad SB (2017). Growth of copper sulfate pentahydrate single crystals by slow evaporation technique. J. Adv. Phys..

[37] Wang Y, Lany S, Ghanbaja J, Fagot-Revurat Y, Chen YP, Soldera F, Horwat D, Mücklich F, Pierson JF (2016). Electronic structures of Cu_2_O, Cu_4_O3, and CuO: a joint experimental and theoretical study. Phys. Rev. B..

[38] Khumalo FS, Huges HP (1980). Vacuum-ultraviolet reflectivity of some α-Na_2_FeO_2_ layer-type compounds. Phys. Rew. B..

[39] Abramova GM, Petrakovskii GA (2006). Metal-insulator transition, magnetoresistance, and magnetic properties of 3d-sulfides (Review). Low Temp. Phys..

